# Supplementary material for: MetaRibo-Seq measures translation in microbiomes
Source: Nat Commun. 2020 Jun 29;11:3268. doi: 10.1038/s41467-020-17081-z (PMC7324362; doi:10.1038/s41467-020-17081-z)
Supplement: Supplementary file 10 — Supplementary Data 7 [file 41467_2020_17081_MOESM10_ESM.zip › File2/Confidence_VeryHigh_Taxonomy/72160_out.krona.html]

Javascript must be enabled to view this page.

members
magnitude
magnitudeUnassigned
count
unassigned
taxon
rank

72160\_out

12

superkingdom
2
11

11
phylum
1239

class
186801
10

186802
order
10

186803
family
2

841
genus
1

2049040

SRS076929\_contig\_number\_contig-100\_758.121833
species
1

1898203

SRS049995\_contig\_number\_27321
species
1

family
541000
8

1263
genus
8

species
2293225

SRS077552\_contig\_number\_25980
1

7
species
165186

SRS014459\_contig\_number\_37293SRS014855\_contig\_number\_15586SRS015065\_contig\_number\_23548SRS016095\_contig\_number\_23669SRS018623\_contig\_number\_contig-100\_1010.133139SRS020869\_contig\_number\_33160SRS050925\_contig\_number\_31138

1
species

SRS015854\_contig\_number\_944
1263031

1

SRS019068\_contig\_number\_contig-100\_3588.489078
